# Chicks produce consonant, sometimes jazzy, sounds

**DOI:** 10.1098/rsbl.2024.0374

**Published:** 2024-09-25

**Authors:** Gianmarco Maldarelli, Andrea Dissegna, Andrea Ravignani, Cinzia Chiandetti

**Affiliations:** ^1^ Department of Life Sciences, University of Trieste, Trieste, Italy; ^2^ Department of Biopsychology, Institute of Cognitive Neuroscience, Faculty of Psychology, Ruhr-Universitat Bochum, Bochum, Germany; ^3^ Comparative Bioacoustics Group, Max Planck Institute for Psycholinguistics, Nijmegen, The Netherlands; ^4^ Center for Music in the Brain, Aarhus University, Aarhus, Denmark; ^5^ Department of Human Neurosciences, Sapienza University of Rome, Rome, Italy

**Keywords:** social building block, innateness, consonance/dissonance, domestic chick, vocalization, musicality

## Abstract

Several animal species prefer consonant over dissonant sounds, a building block of musical scales and harmony. Could consonance and dissonance be linked, beyond music, to the emotional valence of vocalizations? We extracted the fundamental frequency from calls of young chickens with either positive or negative emotional valence, i.e. contact, brood and food calls. For each call, we calculated the frequency ratio between the maximum and the minimum values of the fundamental frequency, and we investigated which frequency ratios occurred with higher probability. We found that, for all call types, the most frequent ratios matched perfect consonance, like an arpeggio in pop music. These music-like intervals, based on the auditory frequency resolution of chicks, cannot be miscategorized into contiguous dissonant intervals. When we analysed frequency ratio distributions at a finer-grained level, we found some dissonant ratios in the contact calls produced during distress only, thus sounding a bit jazzy. Complementing the empirical data, our computational simulations suggest that physiological constraints can only partly explain both consonances and dissonances in chicks’ phonation. Our data add to the mounting evidence that the building blocks of human musical traits can be found in several species, even phylogenetically distant from us.

## 1. Introduction

Why do we enjoy consonant, and sometimes dissonant, sounds? The empiricism of Lockean legacy and the innatism of Platonic stance find one of the most stirring and heated contrasts in one question: why is consonance—i.e. the small-integer ratio relationship between two sound frequencies—often judged pleasurable? Despite culture- and experience-dependent modulations in the appreciation of consonance [1], converging evidence suggests that consonance preference is partly physiologically determined [2,3]. Indeed, while preference can be influenced by musical production of a specific culture, human voice spectra, normalized to fundamental frequency (*f*_0_) and averaged across large databases, exhibit consonant relationships between statistical energy peaks [4]. Similarly, some songbirds produce consonant intervals in their natural songs (e.g. [5]) and music-like precision correlates with male reproductive success [6], indicating that consonant production is tightly linked to a specific selective pressure that extensively exposes individuals to specific harmonic sequences. Mosquitoes also show a comparable selective pressure because, during their ‘love songs’, they converge in harmonic matching [7]. However, mosquitoes do not practice their song, underlining a partial independence between production and training or exposure. Moreover, there is evidence that other non-singing birds prefer consonant sounds from early moments of life, when exposure to consonance has been limited. For instance, newborn domestic chickens preferentially join an artificial social partner emitting consonant melodies [8]. We thus wondered whether the root of such preference lies in natural calls that might be inherently consonant following the idea of consonance as a core feature in communication. To investigate whether consonances also prevail in a non-human, non-singing newborn, we recorded and analysed the main calls of chickens with positive and negative emotional valence. We also considered biological constraints such as the chicks’ range of vocal production and their acoustic discrimination threshold [9] to determine whether chicks could perceive the consonant signature of each call.

## 2. Material and methods

### (a) Animals and rearing conditions

A total of 130 domestic chicks (*Gallus gallus*) Ross 308 broiler (Aviagen) participated in this experiment (females = 63). The eggs were supplied to the laboratory by a local commercial hatchery (Agricola Berica S. C. R. L., Montegalda, Italy). During incubation, the eggs were maintained in an automatically turning incubator in a dark, temperature- and humidity-controlled environment. Once hatched, the chicks were reared for 4 days in pairs in rectangular cages (width, height, depth: 22, 30, 40 cm, respectively) at a controlled room temperature (31°C). The vocalizations were recorded when chicks were between 1 and 4 days old.

### (b) Experimental apparatus and recording procedure

The apparatus consisted of a cylindrical arena (diameter: 60 cm, height: 80 cm); its walls were internally covered with sound-absorbent foam, designed to block external sounds. Calls were recorded via a AKG C1000S Microphone (Wien, Austria) positioned inside the apparatus at a height of 30 cm from the chick’s head. The sound was digitally converted by a Focusrite Scarlett 2i4 audio card (High Wycombe, UK) connected to a computer (sampling rate: 44.1 kHz; amplitude resolution: 16 bits). Only one type of call was recorded from each 4-day-old chick.

**Figure 1.**
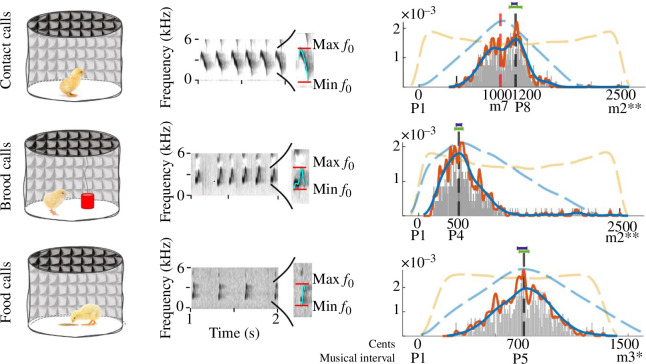
Chicks’ vocalizations: extracting frequency ratios and musical intervals from each type of call. (*a*) The recording conditions. Four-day-old chicks were individually placed within a sound-attenuating circular arena. Each chick was individually recorded for one type of call within the experimental apparatus. We collected contact calls from chicks left alone in the empty arena, brood calls were elicited using an imprinting object hung above the centre of the arena, and for food calls we placed a food dish in the centre of the arena. (*b*) Examples of the spectrogram of chicks’ vocalizations (Praat) highlighting the min and max *f*_0_ (i.e. fundamental frequency) values used to compute frequency ratios. The light blue line indicates the fundamental frequency extracted from the call. (*c*) The distributions of cent ratios across call types. The empirical distributions were obtained from the analysis of 660 contact calls, 486 brood calls and 660 food calls, across 135 chicks. Each distribution is visualized by two kernel density estimations (KDE): one for the global maxima analysis, with a kernel width of 68 cents (the blue-solid line), and one for the local maxima analysis, with kernel width of 10 cents (orange solid line). The histograms show the distribution of frequency ratios without KDE smoothing (bin width = 5 cents). The orange dashed lines show the null distributions, highlighting how the cent ratios would be distributed in the absence of vocal modulation (see electronic supplementary material). The blue dashed lines represent the simulated distributions based on chicks’ vocal range (see electronic supplementary material). The mode of each empirical distribution is shown by a black dashed line. For contact calls, the mode corresponds to 1200 cents, i.e. the octave (p8) in musical notation. In the case of brood and food calls, the modes correspond to 500 and 700 cents, i.e. the perfect fourth (p4) and the perfect fifth (p5), respectively. The violet and green horizontal bars represent the discrimination threshold and the 95% confidence interval, respectively. Finally, the red dashed line marks a dissonant ratio (minor seventh, m7, 1000 cents) as an example of what we predicted to be produced during the emission of contact calls. The number of asterisks indicates the number of octaves above the base interval (p1). One asterisk (*) denotes an increase of one octave (1200 cents) from the base interval, and two asterisks (**) denote an increase of two octaves (2400 cents).

The three types of calls that we analysed were as follows: contact calls, brood calls and food calls. Figure 1*b* contains the spectrographic representation of each of them. These three call types represent major stereotyped vocalizations crucial for communication between a chick and its mother, its imprinting object or another chick. Contact calls are characterized by a descending fundamental frequency and are emitted by chicks when they experience discomfort, especially when they are separated from the hen, their imprinting object or their brood. By contrast, brood calls contain both ascending and descending frequencies and occur under more favourable and mild situations, often when chicks are brooding. Finally, food calls, characterized by ascending frequencies, are typically elicited when chicks identify a profitable food source (for a description of these calls, and of their role in the vocal communication between chicks and hens, refer to [10,11]). These calls are part of a complex vocal code that chickens develop from hatching to adulthood to communicate their needs to other conspecifics (e.g., [12,13]) and to express the positive or negative nature of a situation they are experiencing [14]. The discomfort conditions in which chicks emit contact calls suggest that this call type conveys a negative state to others. This is also supported by the fact that several acoustic parameters of contact calls are linked with the poor welfare of chicks [15]. By contrast, the favourable, rewarding conditions in which brood and food calls are emitted suggest that these two types of calls are linked to a positive state experienced by chicks [10,11].

We stimulated chicks’ production of each call type using a stepwise procedure designed to recreate the natural situation associated with each of them. This procedure was adapted from the stepwise isolation method used in [16], which has proved as a valid method for recording contact and brood calls. We included a further ‘step’ dedicated to the recording of food calls. Specifically, in our procedure, we recorded (i) contact calls from chicks left alone in the empty arena after being separated by their rearing companion and imprinting object; (ii) brood calls elicited via an imprinting object suspended from above in the centre of the arena after the initial isolation; and (iii) food calls elicited by placing a food dish in the centre of the arena after removing the imprinting object (figure 1*a*).

### (c) Acoustic analysis

Audio recordings were manually annotated in Praat v. 6.0.1 [17]. The spectrogram of each recorded audio was visually inspected to detect valid calls. A valid call was processed when it (i) occurred within a sequence of similar calls within 1 s from each other; (ii) fell between the third and third to last position within the call period. We set these two criteria to maximize the chances of analysing calls that are similar and exclude isolated, transitional, ones. Following these criteria, we identified, annotated and saved the onsets and offsets of 660 food calls from 44 chicks, 660 contact calls from 44 chicks, and 486 brood calls from 42 chicks.

For each type of call, we extracted the maximum and minimum values of the fundamental frequency in each call using a custom-written Praat script. To do so, we used the default settings of the *f*_o_ detection function and defined the *f*_0_ range within the 1000–6000 Hz window (the auditory range of chicks extends from 9.1 to 7200 Hz, with peak sensitivity at 2000 Hz [18]). We focused on this *f*_0_ range to include the frequencies that chicks are most sensitive to. Moreover, previous literature and preliminary data indicate that this range is appropriate to include the typical vocalization frequencies of 4-day-old chicks [16]. The time steps were 0.001 s and the size of the analysis window was 3× the period of the minimum frequency. We imported the data in Excel and calculated the cent ratios from the extracted frequencies. Cents are a logarithmic unit of measure for musical intervals. We analysed cent ratios, instead of raw frequency ratios, for three reasons. First, the cents scale provides a normalized measurement system; second, the logarithmic cents scale is designed to reflect human perceptual experiences more accurately than a linear frequency scale; third, cents can be directly related to musical intervals, facilitating the comparison between human music and animal vocalizations. To remap the extracted frequencies in the cents scale, we used the formula: cents ratio = 1200 log²(*b*/*a*), with *b* representing the frequency of the maximum *f*_0_ value within a call and *a* representing the frequency of the minimum *f*_0_ value of the same call (refer to the electronic supplementary material for additional details). We focused on the global minimum and maximum *f*_0_ because they are the only mathematically well-defined values in the function *f*_0_ over time.

### (d) Data analysis

We used Kolmogorov–Smirnov tests to assess the normality and compare the distributions of our empirical cent ratios. Next, we tested whether the most frequent cent ratio inherent in chicks’ call overlapped with a musical interval. We estimated the most frequent cent ratio for each call type from the mode (and its 95% bootstrapped confidence intervals (CI)) of its density distribution. Then, we compared this value with the modes obtained from reference distributions (both null and simulated distributions); these references served as benchmarks to probe the purported mechanisms underlying consonance versus dissonance production.

The null distributions served as a reference for evaluating what the cent ratio distributions would look like under purely random sound production, devoid of any influences that might have affected the cents production of chicks. The simulated distributions, instead, represented the expected distributions of cent ratios which would result from the chicks' vocal range alone. Hence, a significant difference between the empirical and simulated distributions indicates that the empirical distributions differ from randomness over the same range.

We generated three null distributions, one for each call type. To generate a null distribution, we calculated cent ratios using minimum and maximum *f*_0_ values, randomly sampled from two uniform distributions, each extending over the entire vocal range for each call type. Both these distributions encompassed the whole chicks’ frequency range characterizing a specific call type. In essence, this sampling procedure simulated a distribution of the log² ratio between two random variables, both following uniform distributions. The resulting cent ratios were generated under random conditions, within the *f*_0_ range of chicks’ vocal production, without the maximum and minimum values being required to originate from the same call unit.

We generated three additional distributions that served to simulate the influence of chicks’ vocal range on the cent ratios typical of each call type. The first distribution corresponded to the range of minimum *f*_0_ values observed for each call type; the second distribution corresponded to the range of maximum *f*_0_ values observed for the same call; the third distribution corresponded to a random sampling of minimum and maximum *f*_0_ values from the previous two distributions. The resulting cent ratios thereby recreated a scenario in which chicks’ vocal cents production is exclusively a by-product of the vocal extension typical of each call type, independent from individuals’ pairing of minimum/maximum *f*_0_ in our sample of data.

For each distribution type (empirical, null, and simulated) of each call type (contact, brood and food), we estimated the most frequent cent ratio by identifying the mode (global maximum) of its density distribution. To obtain the density distributions, we used kernel density estimation functions (KDE; MATLAB function ’ksdensity’). We set the kernel width equal to the cent ratio corresponding to the just noticeable difference (JND) of frequencies in the chicken; in fact, two frequencies within the JND range would be indistinguishable for the chicken. Based on Gray & Rubel [19], we estimated a JND value of 68 cents (see the electronic supplementary material for more details).

To test the difference between the mode of the distributions, we computed a 95% CI by repeatedly bootstrapping (10 000 times) the mode from each distribution (see the percentile bootstrap method [20]). We rejected the null hypothesis of equivalence if the mode of the null or simulated distributions fell beyond this interval.

In addition, we tested whether the mode of the empirical distribution matched one of the musical intervals of equal temperament tuning and whether chicks could reliably discriminate that interval from neighbouring musical intervals. In particular, we computed a discrimination interval for the mode, with the lower and upper bounds of the interval equal to the mode ± the JND/2. We considered the mode distinguishable from the neighbouring musical intervals if their corresponding cent value fell beyond this interval.

Finally, we searched for other prominent cent ratios produced. To this aim, we tested the presence of smaller peaks (local maxima) in our empirical distributions. Here, the kernel width was 10 cents to reduce the smoothing and increase the resolution of the ratio distributions. These local maxima were identified using the MATLAB function ‘findpeaks’. More details on this analysis can be found in the electronic supplementary material.

## 3. Results

The ratios for each call type did not exhibit a uniform distribution. Instead, all call types’ distributions displayed a peak that aligned with perfect consonance (figure 1*c*, black dotted line). The three call distributions differed from each other (two-sample Kolmogorov–Smirnov test: all *D *> 0.34, *p *< 0.001, see electronic supplementary material, table S1). The most represented cent ratio for contact calls was 1214 (95% CI 1135–1295, discrimination threshold 1166–1234), with the closest musical interval represented by the *octave* (1200 cents). The most represented cent ratio for brood calls was 499 (95% CI 447–551, discrimination threshold 466–534), with the closest musical interval represented by the *perfect fourth* (500 cents). Finally, the most represented cent ratio for brood calls was 715 (95% CI 681–749, discrimination threshold 666–734), with the closest musical interval represented by the *perfect fifth* (700 cents). These musical intervals strongly differ from those predicted by the null distributions, which consisted of a flat probability of ratios with two peaks at the edges (figure 1*c*, orange dashed lines; refer to the electronic supplementary material for details).

In order to verify whether the main consonance shown in the global maxima analysis is a solid feature of the distribution regardless of the specific parameters used for the statistical analysis, we ran the same analysis at different values of the KDE smoothing (range from 10 to 100 cents, steps of 10 cents) and extracted the corresponding global maxima. The results show that, for all call types, the global maxima are consistently consonant regardless of the KDE smoothing (see electronic supplementary material).

Next, we employed a finer-grained binning to more accurately detect secondary, smaller peaks, which may represent additional prominent cent ratios within the empirical distributions. We found, in our empirical distributions, additional musical intervals that chicks produced above chance level (see the analysis of local maxima in the electronic supplementary material). These occurred in both food (with two additional cent ratios being significant and matching perfect consonance) and contact calls (with two additional cent ratios being significant and matching dissonance), but never in the brood calls. This result showed that contact calls feature dissonant intervals, but they are less prominent in chicks’ vocal production than expected solely on the basis of their vocal range.

The most frequent cent ratios in the simulated distributions of brood and food calls (517 and 689 cents) matched the musical intervals empirically observed, namely the perfect fourth and the perfect fifth, respectively. The most represented cent ratio resulting from the simulated distribution of contact calls was 1027 corresponding to the dissonant minor seventh. This confirms that contact calls can be expected to contain dissonant intervals, considering the *f*_0_ range of this call type, although these dissonant intervals are less frequent than consonant ones.

## 4. Discussion

This study reveals a prevalent occurrence of perfect consonance across all types of chicks’ calls, supporting the notion that consonant sounds are inherently present in animal communication [3,4]. Given their phylogenetic commonness, consonant sounds could serve as one of the fundamental building blocks that, together with basic visual mechanisms, aid in distinguishing animate objects within the acoustic domain, marking the presence of living entities amidst other natural sounds [21].

Our results also reveal the abundance of consonant sounds in calls associated with positive valence, like food and brood calls, emitted when chicks interact with companions or forage safely. This suggests the potentially crucial role of consonant sounds, particularly in affiliative interactions, as beneficial in strengthening mutual connections—a positive effect similar to that of human prosody and music implementing consonances [22,23].

However, our simulations also predicted the presence of dissonant sounds in contact calls. Indeed, such dissonant component in chicks’ vocal production was notably found in contact calls upon a finer level of analysis. As a result, contact calls might appear more dissonant due to several production factors, including vocal motor control or emotional modulation linked to negative contexts [22], such as attempts to rejoin a companion. Despite the presence of dissonant sounds, the fundamental consonant nature of this call type remains dominant at a statistical level. The results of the simulation do not alter the basic conclusion that chicks’ calls tend to emphasize consonant intervals between maximum and minimum *f*_0_ values. Among the periodic frequencies emitted in vocalizations, consonant ratios prevail over dissonant ones and represent the principal feature characterizing the spectral range of a living being. In other words, consonance might demark the presence of another living being. Conversely, other periodic sounds, such as dissonances, would emphasize the peculiar condition of a distressed individual struggling to locate conspecifics.

Considering the physiological range of *f*_0_s in chicks’ vocalizations, which indicates a consonant profile as typical, especially in emotionally positive emitted calls, and the chick auditory system’s discrimination of these intervals [9], it is plausible to assume that chicks possess a neural mechanism for detecting consonance, allowing recognition of conspecifics.

In other words, chicks’ production of consonant and dissonant musical intervals may respond to specific social and communicative purposes, linked, e.g. to the emotional context of emission. Small-integer ratios compatible with consonance that we found as a social sound signal may also facilitate memory and recall of the location of conspecifics. These are not mutually exclusive interpretations and dovetail with the idea of biological underpinnings for consonance appreciation. Indeed, there is a correspondence between our simulated cent ratios and the empirical ones, highlighting that what we sampled could be expected considering the *f*_0_ range of this species. The fact that the general *f*_0_ range of a call is bound at consonant musical intervals suggests that consonance might have been naturally selected for communication purposes.

Our findings thus highlight chicks’ biological predisposition to produce, and potentially recognize, a building block of human music: consonance. Indeed, the neural machinery for consonance detection might allow it to generalize its appreciation to other sounds, including artificial sounds with a consonant profile [8]. Moreover, the dissonance arising during negative conditions, such as distress calls while seeking conspecifics and to alleviate loneliness, echoes the unresolved tension typical of the dissonant sevenths in jazz music—the same dissonant seventh we find in the data. This unresolved tension typically moves towards consonance, evoking a sense of heightened suspense in the listener due to delayed resolution. Therefore, the present, albeit less frequent, dissonance may have evolved to prompt searching behaviour and lay the foundation for preferences towards mild dissonance [24] and specific musical styles, which can be amplified through exposure and practice.

To conclude, our data add to converging evidence that the building blocks of musicality can be found across multiple species, in this case even in a bird that does not sing or learn sounds. Here, consonance may act as a biological signpost to signal the presence of another living being. This could explain why newborn chicks like [8] and produce consonant sounds.

## Data Availability

The dataset supporting this article has been uploaded as part of the electronic supplementary material [25].
